# Combined effects of body position and sleep status on the cardiorespiratory stability of near-term infants

**DOI:** 10.1038/s41598-018-27212-8

**Published:** 2018-06-11

**Authors:** Yoshihisa Oishi, Hidenobu Ohta, Takako Hirose, Sachiko Nakaya, Keiji Tsuchiya, Machiko Nakagawa, Isao Kusakawa, Toshihiro Sato, Toshimasa Obonai, Hiroshi Nishida, Hitoshi Yoda

**Affiliations:** 1 Department of Pediatrics, Japanese Red Cross Medical Center, 4-1-22 Hiroo, Shibuya-ku, Tokyo 150-8935 Japan; 2 Department of Neonatology, Toho University Graduate School of Medicine, 6-11-1 Omorinishi, Ota-ku, Tokyo 143-8541 Japan; 3 Department of Pyschophysiology, National Institute of Mental Health, National Center of Neurology and Psychiatry, 4-1-1 Ogawa-higashi-cho, Kodaira, Tokyo 187-8553 Japan; 4 Department of Psychiatry, Asai Hospital, 38-1 Togane, Chiba, 283-0062 Japan; 5 Department of Pediatrics, St. Luke’s International Hospital, 9-1 Akashi-cho, Chuo-ku, Tokyo 104-8560 Japan; 6 Department of Neonatology, School of Medicine, Faculty of Medicine, Toho University, 6-11-1 Omorinishi, Ota-ku, Tokyo 143-8541 Japan; 7 Department of Global Marketing, Unicharm Corporation, 3-5-27 Mita, Minato-ku, Tokyo 108-8575 Japan; 8 Department of Pediatrics, Tama-Hokubu Medical Center, Tokyo Metropolitan Health and Medical Treatment Corporation, 1-7-1 Aoba-chou, Higashimurayama City, Tokyo 189-8511 Japan; 9 Department of Maternal and Neonatal Medicine, Tokyo Women’s Medical College, 8-1 Kawada-cho, Shinjuku-ku, Tokyo 162-0054 Japan

## Abstract

The purpose of this study was to determine the effects of body position (prone, supine and lateral) together with sleep status (wake and sleep) on the cardiorespiratory stability of near-term infants. A total of 53 infants (gestational age at birth 33.2 ± 3.5 weeks; birth weight 1,682 ± 521 g; gestational age at recording 38.6 ± 2.1 weeks; weight at recording: 2,273 ± 393 g) were monitored for 24 hours for clinically significant apnea (>15 seconds), bradycardia (<100 bpm), and oxygen desaturation (SpO_2_ < 90%) in alternating body positions (prone, supine and lateral) by cardiorespiratory monitors and 3-orthogonal-axis accelerometers. Sleep status of the infants was also continuously monitored by actigraphs. No apnea was observed. During wake, severe bradycardia was most frequently observed in the lateral position while, during sleep, severe bradycardia was most frequently observed in the supine position. Desaturation was most frequently observed in the supine and lateral positions during both wake and sleep. Our study suggests that the cardiorespiratory stability of infants is significantly compromised by both body position and sleep status. During both wake and sleep, prone position induces the most stable cardiorespiratory functions of near-term infants.

## Introduction

The effects of body position of infants on pathological and physiological processes have been closely investigated during the last decade, ever since a continuous decrease in the occurrence rate of sudden infant death syndrome (SIDS) was reported following the introduction of non-prone positioning for resting infants of less than one year of age, as recommended by the American Academy of Pediatrics in 1992^1–3^. In clinical practice, however, improved oxygenation and decreased incidence of gastroesophageal reflux have been also reported for infants in the prone position and there is an ongoing debate on whether the supine position is suitable for near-term infants to achieve stable cardiorespiratory functions^4–7^. So far, no study has examined the effects of sleep status (wake and sleep) with body position (prone, supine and lateral) on infants’ cardiorespiratory stability even though SIDS may happen not only during sleep but also during wake periods. In this study, using actigraphs and 3-orthogonal-axis accelerometers, we measured sleep status and body position of infants simultaneously for 24 consecutive hours. The aim of our study was to objectively evaluate the combined effects of body position and sleep status on the cardiorespiratory stability of near-term infants using automatic monitoring systems for all related parameters and to determine which body position is the most appropriate for achieving stable cardiorespiratory functions for near-term infants.

## Results

**Figure 1:**
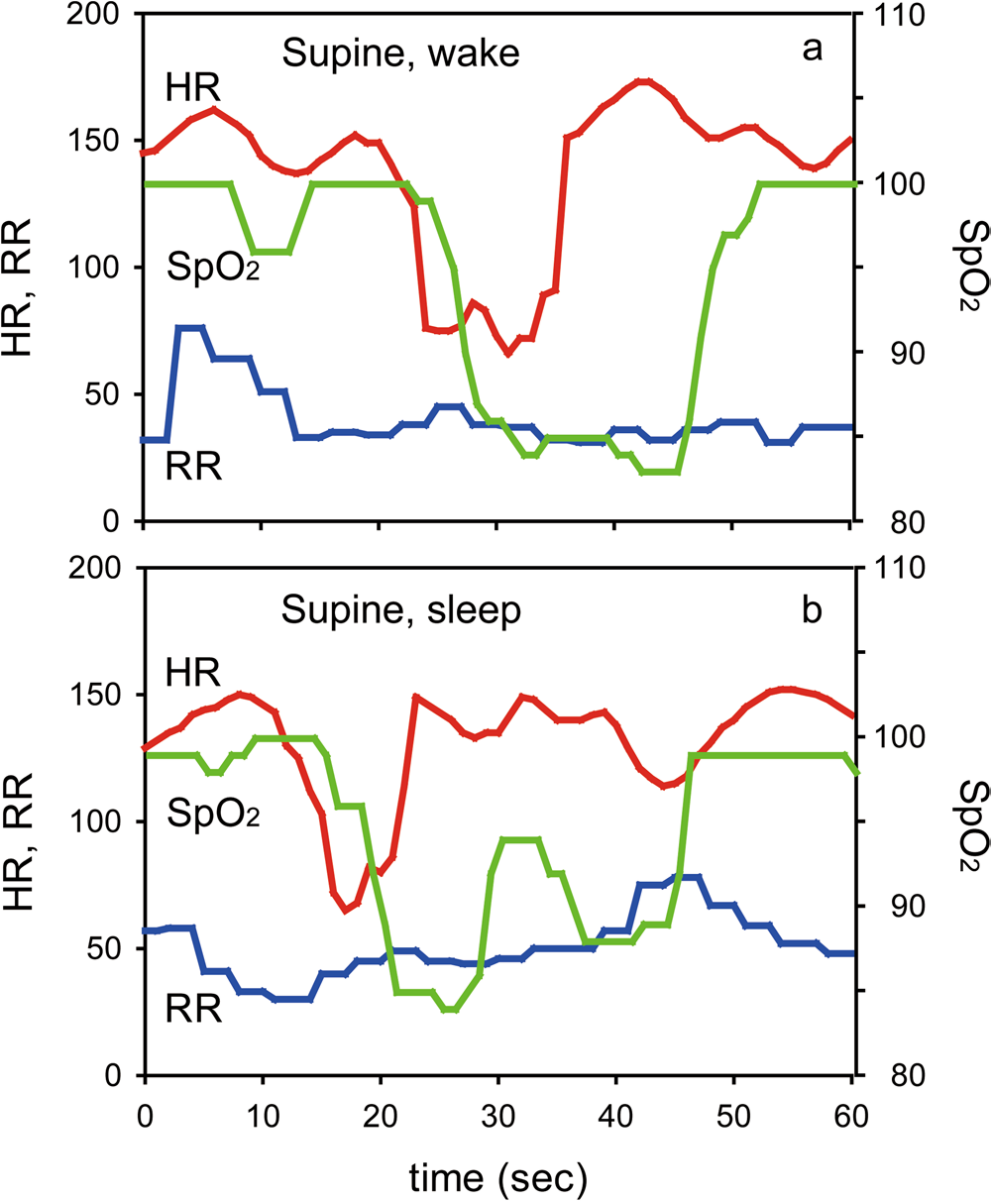
Bradycardia and desaturation in the supine position. (**a**) Typical traces of heart rate (HR: red), respiratory rate (RR: blue), and SpO_2_ (green) during wake in the supine position. (**b**) Typical traces of HR, RR, and SpO_2_ during sleep in the supine position. An episode of bradycardia (<100 bpm) followed by desaturation (<90%) was observed in each case.

**Figure 2:**
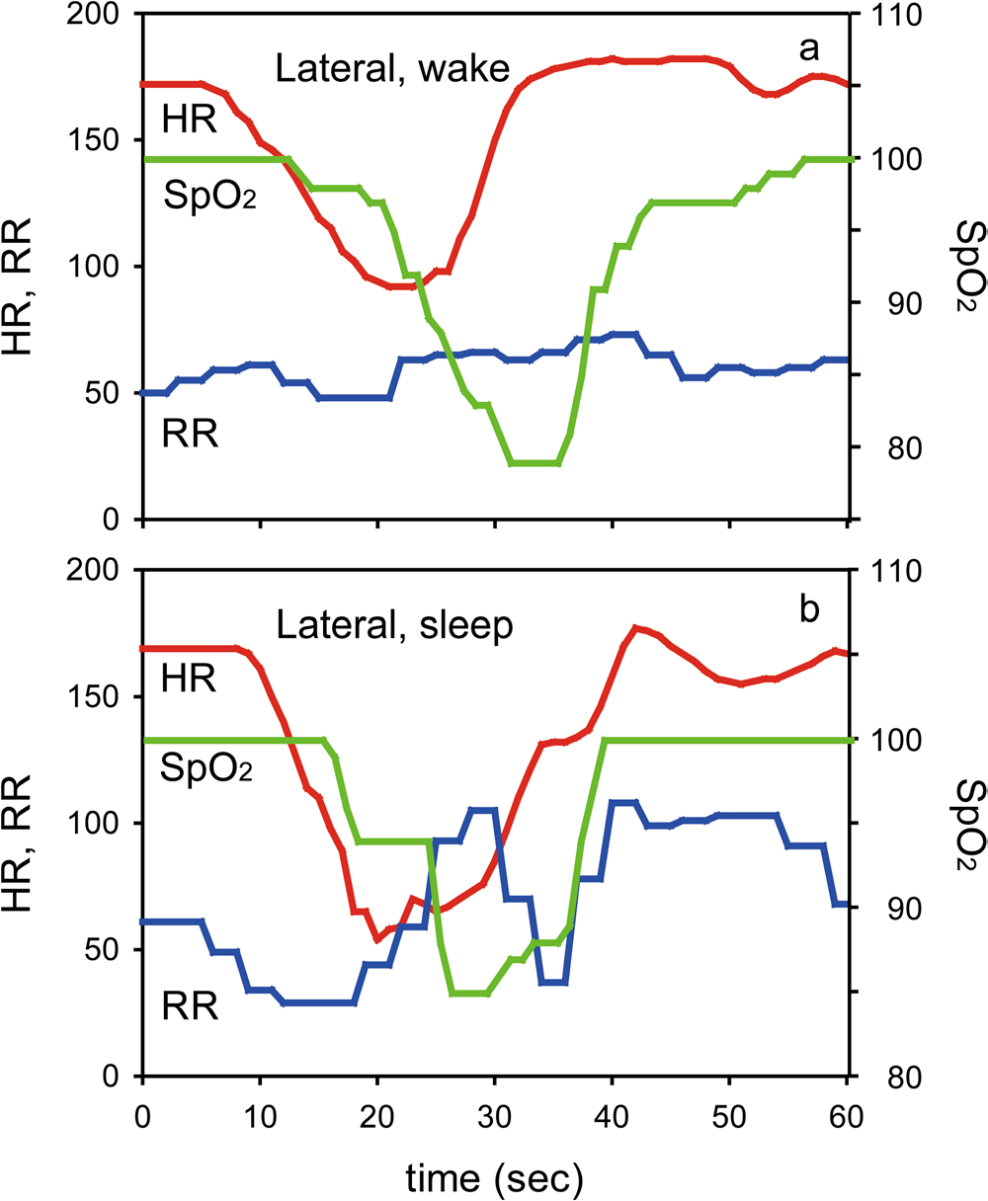
Bradycardia and desaturation in the lateral position. (**a**) Typical traces of heart rate (HR: red), respiratory rate (RR: blue), and SpO_2_ (green) during wake in the lateral position. (**b**) Typical traces of HR, RR, and SpO_2_ during sleep in the lateral position. An episode of bradycardia (<100 bpm) followed by desaturation (<90%) was observed in each case.

**Figure 3:**
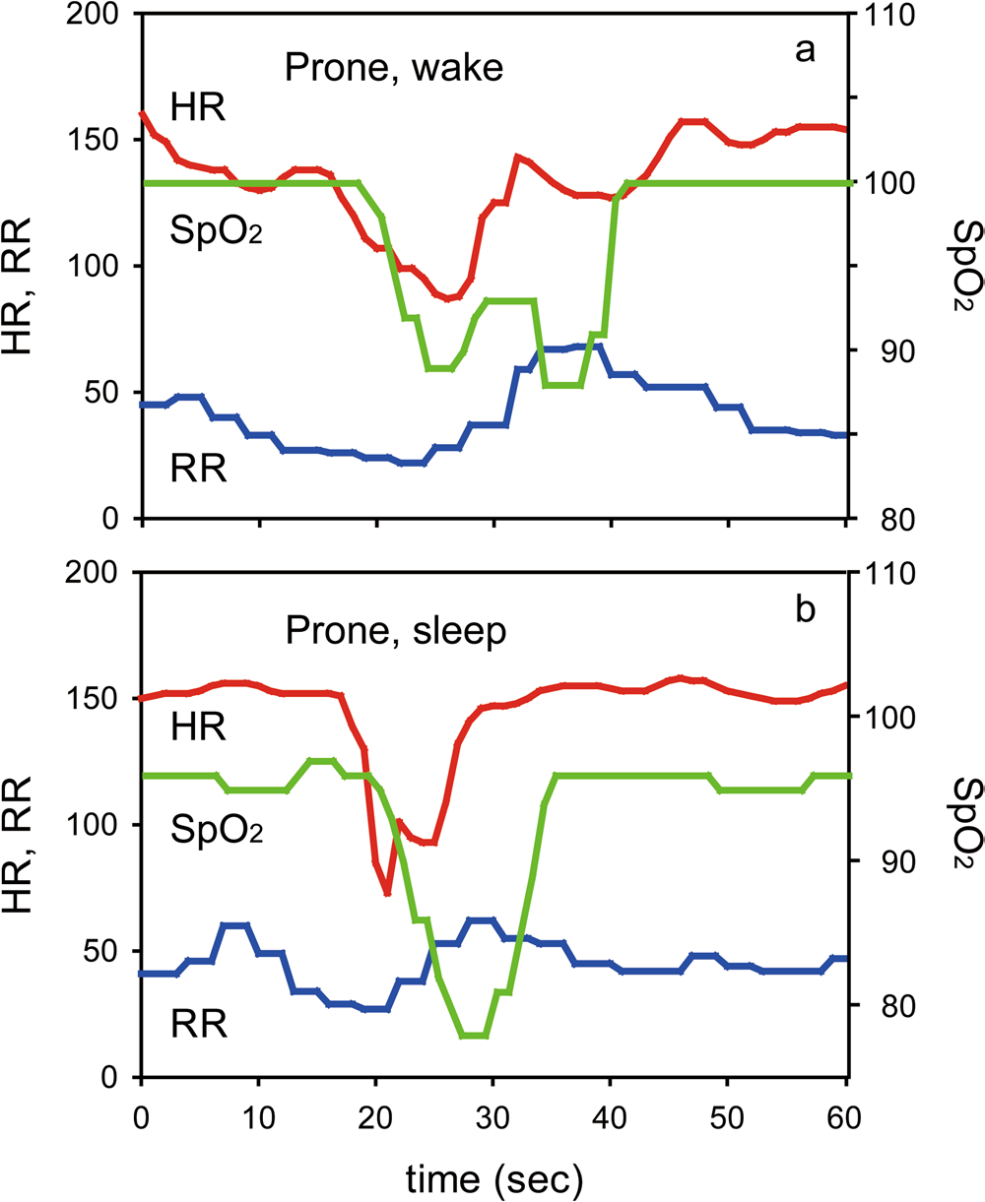
Bradycardia and desaturation in the prone position. (**a**) Typical traces of heart rate (HR: red), respiratory rate (RR: blue), and SpO_2_ (green) during wake in the prone position. (**b**) Typical traces of HR, RR, and SpO_2_ during sleep in the prone position. An episode of bradycardia (<100 bpm) followed by desaturation (<90%) was observed in each case.

A total of 53 infants (gestational age at recording: 38.6 ± 2.1 weeks and weight at recording: 2,273 ± 393 g) were monitored for 24 hours for apnea, bradycardia, and oxygen desaturation in alternating body positions (prone, supine and lateral) by cardiorespiratory monitors and accelerometers (Table 1, Figs 1–3). Sleep status (wake and sleep) of the infants was also continuously monitored by actigraphs. A total of 2,112 seconds (39.8 ± 53.5 seconds/infant/day) of bradycardia (<100 beats per minute: bpm) and 20,646 seconds (389.5 ± 372.1 seconds/infant/day) of oxygen desaturation (SpO_2_ < 90%) were considered valid. No clinically significant episodes of apnea (>15 seconds) were confirmed in any infants during observation. In addition, there was also no clinically significant episode of apnea >10 seconds or >5 seconds in any infants during observation. No specific body position was found to be statistically different in occurrence to the others after feeding (F-test followed by residual analysis, p > 0.05).

**Table 1:** Characteristics of participants.

Gestational age at birth (weeks), mean ± s.d.	33.2 ± 3.5
Birth weight (g), mean ± s.d.	1,682 ± 521
No. of infants (girls: boys)	53 (29:24)
Gestational age at recording (weeks), mean ± s.d.	38.6 ± 2.1
Weight at recording (g), mean ± s.d.	2,273 ± 393

### Effects of body position on cardiorespiratory stability of infants

The overall incidence of bradycardia was greatest in the supine position (occurrence rate 0.062%, 17.1 ± 25.7 seconds/infant/day), followed by the lateral position (occurrence rate 0.056%, 14.5 ± 25.6 seconds/infant/day), then followed by the prone position (occurrence rate 0.041%, 8.2 ± 17.8 seconds/infant/day). These differences were statistically significant (supine > lateral > prone; F-test followed by residual analysis, p < 0.05; Table 2, Supplementary Table S1). The overall incidence of oxygen desaturation was greatest in the supine position (occurrence rate 0.666%, 185.2 ± 213.6 seconds/infant/day), followed by the lateral position (occurrence rate 0.574%, 150.0 ± 174.8 seconds/infant/day), then followed by the prone position (occurrence rate 0.269%, 54.3 ± 68.3 seconds/infant/day). However, no statistical difference between the supine and lateral positions was detected and the prone position had a significantly lower frequency of desaturation compared to the supine and prone positions (supine, lateral > prone; F-test followed by residual analysis, p < 0.05; Table 3, Supplementary Table S2).

**Table 2:** Total sum durations of bradycardia of all infants (n = 53) among the three body positions throughout the 24-hour recordings.

	Supine	Lateral	Prone
HR < 100 bpm (sec)	908	770	434
HR ≧ 100 bpm (sec)	1,460,369	1,383,575	1,070,340
Occurrence rate	0.062%	0.056%	0.041%

**Table 3:** Total sum durations of desaturation of all infants (n = 53) among the three body positions throughout the 24-hour recordings.

	Supine	Lateral	Prone
SpO_2_ < 90 (sec)	9,815	7,951	2,880
SpO_2_ ≧ 90 (sec)	1,463,533	1,376,431	1,067,899
Occurrence rate	0.666%	0.574%	0.269%

### Effects of sleep status on cardiorespiratory stability of infants

Significant influence of sleeping status was noted in the overall incidence of bradycardia, indicating that infants during wake (occurrence rate 0.076%, 22.2 ± 31.3 seconds/infant/day) experienced statistically greater incidence of bradycardia than during sleep (occurrence rate 0.040%, 17.7 ± 36.2 seconds/infant/day) (F-test, p < 0.05; Table 4, Supplementary Table S3). The overall incidence of oxygen desaturation was also statistically greater during wake (occurrence rate 0.964%, 283.0 ± 335.4 seconds/infant/day) than during sleep (occurrence rate 0.238%, 106.6 ± 149.5 seconds/infant/day) (F-test, p < 0.05; Table 5, Supplementary Table S4).

**Table 4:** Total sum durations of bradycardia of all infants (n = 53) between wake and sleep throughout the 24-hour recordings.

	Wake	Sleep
HR < 100 bpm (sec)	1,174	938
HR ≧ 100 bpm (sec)	1,543,015	2,371,269
Occurrence rate	0.076%	0.040%

**Table 5:** Total sum durations of desaturation of all infants (n = 53) between wake and sleep throughout the 24-hour recordings.

	Wake	Sleep
SpO_2_ < 90% (sec)	14,997	5,649
SpO_2_ ≧ 90% (sec)	1,541,307	2,366,556
Occurrence rate	0.964%	0.238%

### Combined effects of both body position and sleep status on cardiorespiratory stability of infants

As above mentioned, since no episodes of apnea were observed in infants, the combined effects of body position and sleep status on apnea were not analyzed.

Regarding the incidence of bradycardia, significant difference from position was noted both during wake and sleep. However, the body position differently affected the incidence of bradycardia, depending on sleep status. During wake, the overall incidence of bradycardia was greatest in the lateral position (occurrence rate 0.083%, 8.8 ± 16.8 seconds/infant/day), followed by the supine position (occurrence rate 0.078%, 10.2 ± 13.7 seconds/infant/day), then followed by the prone position (occurrence rate 0.058%, 3.1 ± 12.0 seconds/infant/day). Significant differences among the three body positions were statistically significant during wake (lateral > supine > prone; F-test followed by residual analysis, p < 0.05; Table 6, Supplementary Table S5). In contrast, during sleep, the overall incidence of bradycardia was greatest in the supine position (occurrence rate 0.048%, 6.9 ± 17.5 seconds/infant/day), followed by the lateral position (occurrence rate 0.037%, 5.7 ± 16.0 seconds/infant/day), then followed by the prone position (occurrence rate 0.034%, 5.1 ± 12.7 seconds/infant/day). Significant differences among the three body positions were also statistically significant during sleep (supine > lateral > prone; F-test followed by residual analysis, p < 0.05; Table 7, Supplementary Table S6). This indicates that infants’ cardiac stability may be affected by the combined influence of body position and sleep status since the positions that lead to the highest incidence of bradycardia differed between sleep and wake.

**Table 6:** Total sum durations of bradycardia of all infants (n = 53) among the three body positions during wake.

	Supine	Lateral	Prone
HR < 100 bpm (sec)	543	467	164
HR ≧ 100 bpm (sec)	697,452	564,293	281,270
Occurrence rate	0.078%	0.083%	0.058%

**Table 7:** Total sum durations of bradycardia of all infants (n = 53) among the three body positions during sleep.

	Supine	Lateral	Prone
HR < 100 bpm (sec)	365	303	270
HR ≧ 100 bpm (sec)	762,917	819,282	789,070
Occurrence rate	0.048%	0.037%	0.034%

In contrast to the above-mentioned significant difference in rates of bradycardia due to sleep status, no statistical difference in the incidence of oxygen desaturation due to sleep status was found. During wake, the overall incidence of oxygen desaturation was greatest in the supine position (occurrence rate 1.034%, 138.5 ± 177.2 seconds/infant/day), followed by the lateral position (occurrence rate 1.003%, 106.8 ± 156.8 seconds/infant/day), then followed by the prone position (occurrence rate 0.708%, 37.6 ± 63.9 seconds/infant/day). However, no statistical difference between the supine and lateral positions was detected and the prone position had the least significantly frequent incidence of oxygen desaturation compared to the supine and prone positions (supine, lateral > prone; F-test followed by residual analysis, p < 0.05; Table 8, Supplementary Table S7). During sleep, the overall incidence of oxygen desaturation was greatest in the supine position (occurrence rate 0.324%, 46.6 ± 88.4 seconds/infant/day), followed by the lateral position (occurrence rate 0.279%, 43.2 ± 80.4 seconds/infant/day), then followed by the prone position (occurrence rate 0.112%, 16.8 ± 32.1 seconds/infant/day). However, no statistical difference between the supine and lateral positions was detected and the prone position had the least significantly frequent incidence of oxygen desaturation compared to the supine and prone positions (supine, lateral > prone; F-test followed by residual analysis, p < 0.05; Table 9, Supplementary Table S8). The fact that there was a tendency for no difference in occurrence of desaturation between wake and sleep indicates that infants’ respiratory stability may not be affected by a combined influence of body position and sleep status and may only be influenced by body position.

**Table 8:** Total sum durations of desaturation of all infants (n = 53) among the three positions during wake.

	Supine	Lateral	Prone
SpO_2_ < 90% (sec)	7,343	5,662	1,992
SpO_2_ ≧ 90% (sec)	702,742	559,118	279,447
Occurrence rate	1.034%	1.003%	0.708%

**Table 9:** Total sum durations of desaturation of all infants (n = 53) among the three positions during sleep.

	Supine	Lateral	Prone
SpO_2_ < 90% (sec)	2,472	2,289	888
SpO_2_ ≧ 90% (sec)	760,791	817,313	788,452
Occurrence rate	0.324%	0.279%	0.112%

## Discussion

The question addressed by the present study is whether the cardiorespiratory stability of near-term infants of around 38 weeks’ gestational age before discharge is affected significantly by body position and/or sleep status. We found three answers to this question.

First, the combined effects of both body position and sleep status affected the occurrence rate of bradycardia. During “wake”, episodes of bradycardia were most frequently induced by the “lateral” position followed by the supine position and then prone position, while during “sleep”, more episodes of bradycardia were induced in the “supine” position followed by the lateral position and then prone position. The reason why “lateral” and “supine” positions induced more episodes of bradycardia during “wake” and “sleep” respectively remains unclear but vagal tone affected by both body positioning and sleep status may cause such difference. Edner *et al*. reported that head positioning such as head upright tilt, which also increases sympathetic tone, can decrease the heart rate of infants of around 9 weeks^8^. Our result is consistent with Goto’s study, in which more awakenings and heart rate variability were found in 36-week-postconceptional age preterm infants in supine position during sleep^9^. However, our data is inconsistent with Keene’s study, in which no difference in apnea, bradycardia, or desaturation was found between the supine or prone position^10^. The difference may come from the monitoring system and experimental design. Unlike our 24-hour recording in a natural setting, Keene routinely changed the infants’ body position every 6 hours during the 24-hour observation period^10^ and did not evaluate sleep or wake status with polysomnography or actigraphs. Goto *et al*. did monitor infants for 6 hours with polysomnography and videos^9^ but did not do so for 24 hours. Our study is the first to report the combined effects of both body position and sleep status on infant bradycardia in a natural setting, monitoring 24-hour physiological parameters with an accelerometer and an actigraph.

Second, body position affected the occurrence rate of desaturation but sleep status did not. During both “wake” and “sleep”, episodes of desaturation were most frequently induced by the “supine” and “lateral” positions followed by the prone position. Our result implies that, unlike bradycardia, physiological mechanisms based on sleep status, such as vagal tone, do not affect infant breathing. The present result is consistent with the improved oxygenation in the prone position that has been reported by several groups of investigators^5–7,11^. Poets *et al*. also reported that infants of approximately 39 weeks gestational age had more desaturation events while sleeping in the supine position^12^. An increase in arterial oxygen tension has been demonstrated in the prone position in adult patients requiring ventilatory assistance^5^. Subsequent studies in preterm infants have confirmed similar increases in arterial oxygen tension in the prone position compared with the supine position^6,13^. Asynchronous chest wall movement in the supine position has been demonstrated in preterm infants^14^. This decreased asynchronous chest wall movement in the prone position may enhance the ventilation/perfusion ratio, resulting in improved oxygenation^6^.

Third, stable cardiorespiratory function without bradycardia and desaturation was maintained more by prone position than by either supine or lateral position. In particular, our data indicate that “lateral” position is not necessarily a safe body position, and led to more bradycardia and desaturation than prone position. The result contradicts published data and anecdotal evidence that states that the lateral position can decrease risk of choking^15^, but does suggest that lateral position should be avoided to protect infants from the risk of bradycardia and desaturation. In contrast to the recommendation by the American Academy of Pediatrics (AAP)^2^ that infants be placed in non-prone position every sleep, prone position can be more beneficial for achieving stable cardiorespiratory function of near-term infants in early developmental stages in the Growing Care Units (GCU) before discharge if they are carefully monitored with vital-sign monitoring systems.

Three concerns in this study warrant consideration. First, we did not use polysomnography to evaluate the sleep status of infants. Although polysomnography is the golden standard for analyzing sleep status objectively, the actigraphy device has been reported to be a reliable method for determining sleep in infants around term-equivalent age^16^. Second, since the present study mainly deals with preterm infants, the results may not be directly applied to the cardiorespiratory response of full-term neonates, and a difference in cardiorespiratory stability affected by body positioning and sleep status may exist between preterm and full-term infants. Even so, a similar previous study reported the same results with full-term neonates, namely, that the prone position produces a more stable cardiorespiratory response compared to the supine position^13,17,18^. Third, because of the relatively small sample size (n = 53), this study did not fully perform analysis on sex difference in cardiorespiratory monitor, body position monitor, or actigraph variables. Since the study population is also not well-balanced between girls (n = 29) and boys (n = 24), a study population with a larger sample size will be required to make a satisfactory statistical analysis on the effect of sex difference on infants’ cardiorespiratory variables in any future studies.

The present study demonstrated that prone position results in a lower frequency of clinically significant episodes of bradycardia and/or desaturation in near-term infants of approximately 38 weeks of gestational age during both wake and sleep than supine or lateral position. At present, according to the recommendation against SIDS, healthy preterm and full-term infants before discharge are usually placed in the supine or lateral position, even though there is a reluctance to do so as medical staff often notice better oxygen saturation in infants placed in the prone position. The present study objectively reconfirmed the same points that near-term infants have more stable cardiorespiratory functions in prone position and proposed a possible necessity of searching the best timing of beginning the exposure of the supine position to near-term infants to prevent SIDS. In addition, our study also indicates that it may be safer to avoid putting near-term infants in the lateral position to reduce the risk of bradycardia and desaturation.

## Methods

### Subjects

53 infants (29 girls and 24 boys) were studied. All infants were born at 33.2 ± 3.5 weeks’ gestational age (WGA), with birth weights (1,682 ± 521 g). Apgar score were 4 to 10 (median 9) at 5 minutes. Infants were studied approximately one week before discharge (38.6 ± 2.1 WGA; weights 2,273 ± 393 g). All infants were clinically stable at the time of observation. Their characteristics are summarized in Table 1. Infants were not studied for at least four weeks following extubation, continuous positive airway pressure, oxygen inhalation therapy, or a work-up for sepsis. They were excluded from the study if they had any condition that precluded them from being placed in either the prone or supine position (e.g., gastroschisis and meningomyelocele, respectively). A total of 13 infants were receiving methylxanthine, and 3 infants were receiving both methylxanthine and doxapram for the treatment of apnea. However, no infant was treated with methylxanthine or doxapram within the 24-hour period before the observation started. A total of 7 infants had been treated for respiratory distress syndrome with mechanical ventilation and surfactant administration. No infant had bronchopulmonary dysplasia. The ethics committees of the Japanese Red Cross Medical Center approved the study protocol (ethical approval number 716) and all procedures were carried out in accordance with the approved guidelines. Written informed consent was obtained from the parents.

### Study design

Infants were naturally observed in the Growing Care Unit (GCU), which is used for supporting preterm and term infants as they mature before discharge. Each infant was placed into the prone, supine or lateral position, according to their medical or care circumstances, and observed in that position for a continuous 24-hour period with a monitoring system which measured heart rate, respiration rate, oxygen saturation and position. Infants in the prone position were placed with their face to the side, whereas those in the supine position were allowed to assume their natural position. Infants in the lateral position were placed either right-side or left-side upward to prevent esophageal reflux.

### Data collection

Heart rate, respiration rate and oxygen saturation were monitored using standard paste-on electrodes connected to a cardiorespiratory monitor with event-recording capability (BSM-6000; Nihon Kohden Inc., Tokyo, Japan). Event recordings were triggered by any episode of apnea lasting at least 15 seconds, a heart rate of <100 beats per minute (bpm), or by oxygen saturation of <90%. Two investigators reviewed all recorded waveforms to determine their validity, excluding false events from further analysis. Any periods of medical or nursing interventions or parental interactions were noted and removed from analyses. Because hypopneic episodes could not be distinguished from obstructed breaths based on impedance monitoring, only episodes of central apnea were analyzed. Oxygen desaturation during apnea and hypoventilation is a gradual event. Therefore, any instantaneous severe drops in oxygen saturation were considered to be artifacts. True episodes of bradycardia were determined by reviewing electrocardiogram waveform during the alarm. Clinically significant episodes were defined as apnea lasting at least 15 seconds, a heart rate of <100 bpm, or oxygen saturation of <90%.

### Wake and sleep assessment

For wake and sleep measurement, we used Actigraphy. Actigraphy is an objective, non-intrusive method for estimating sleep-wake patterns using activity-based monitoring^16^. The Actigraphy device used in the present study was the Actigraph (Micro-mini RC, Ambulatory Monitoring Inc., NY, USA). We attached the Actigraphs to their child’s waist with an adjustable elastic belt for 24 hours. Waist attachment was chosen as we found it less disturbing than wrist or ankle attachment. Motility levels were sampled in the zero-crossing mode in 1-min epochs. The resolution of the Actigraph was set at 0.01 G/s. The activity data recorded by the Actigraph was later downloaded using ACTme software (ver. 3.10.0.3, Ambulatory Monitoring Inc.), and then sleep measures were analyzed using Action-W software (ver. 2.4.20, Ambulatory Monitoring Inc.). During the study, time intervals when the device was removed, for example, during bathing, were recorded in a sleep diary by nurse.

### Body position assessment

The infants’ positions were monitored for 24 hours both by visual observation of nursing staffs and also by a ADXL345 MEMS accelerometer (Analog Devices, MA, USA), with which acceleration of the body can be measured along 3 orthogonal axes. Both the ADXL345 MEMS accelerometer and the above-mentioned Actigraph were attached to child’s waist with the same adjustable elastic belt.

### Statistical analysis

Statistical analyses were performed with SPSS Statistics 21.0 (IBM Corp. Armonk, NY, USA). Statistical significance for differences between prone, supine and lateral positioning in a natural GCU (Growing Care Unit) setting was determined using the F-test followed by residual analysis. A *p* value of <0.05 was considered significant.

## Electronic supplementary material

Supplementary Tables
