# Supplementary material for: Combined effects of body position and sleep status on the cardiorespiratory stability of near-term infants
Source: Sci Rep. 2018 Jun 11;8:8845. doi: 10.1038/s41598-018-27212-8 (PMC5995963; doi:10.1038/s41598-018-27212-8)
Supplement: Supplementary file 1 — Supplementary Tables [file 41598_2018_27212_MOESM1_ESM.docx]

**Combined effects of body position and sleep status on the cardiorespiratory stability of near-term infants**

Yoshihisa Oishi^1, 2^, Hidenobu Ohta^3, 4^*, Takako Hirose^1^, Sachiko Nakaya^1^, Keiji Tsuchiya^1^, Machiko Nakagawa^5, 6^, Isao Kusakawa^5^, Toshihiro Sato^7^, Toshimasa Obonai^8^, Hiroshi Nishida^9^ & Hitoshi Yoda^6^

^1^ Department of Pediatrics, Japanese Red Cross Medical Center, 4-1-22 Hiroo, Shibuya-ku, Tokyo 150-8935, Japan

^2^ Department of Neonatology, Toho University Graduate School of Medicine, 6-11-1 Omorinishi, Ota-ku, Tokyo 143-8541, Japan

^3^ Department of Pyschophysiology, National Institute of Mental Health, National Center of Neurology and Psychiatry, 4-1-1 Ogawa-higashi-cho, Kodaira, Tokyo 187-

8553, Japan

^4^ Department of Psychiatry, Asai Hospital, 38-1 Togane, Chiba 283-0062, Japan

^5^ Department of Pediatrics, St. Luke’s International Hospital, 9-1 Akashi-cho, Chuo-ku, Tokyo 104-8560, Japan

^6^ Department of Neonatology, School of Medicine, Faculty of Medicine, Toho University, 6-11-1 Omorinishi, Ota-ku, Tokyo 143-8541, Japan

^7^ Department of Global Marketing, Unicharm Corporation, 3-5-27 Mita, Minato-ku, Tokyo, 108-8575, Japan

^8^ Department of Pediatrics, Tama-Hokubu Medical Center, Tokyo Metropolitan Health and Medical Treatment Corporation, 1-7-1 Aoba-chou, Higashimurayama City, Tokyo 189-8511

^9^ Department of Maternal and Neonatal Medicine, Tokyo Women’s Medical College, 8-1 Kawada-cho, Shinjuku-ku, Tokyo 162-0054

*Correspondence: Hidenobu Ohta

Address: Department of Pyschophysiology, National Institute of Mental Health, National Center of Neurology and Psychiatry, Kodaira, Tokyo, 187-8553 Japan

Tel. +81-42-341-2712 (ext.6274); Fax +81-42-346-2158

E-mail: hideohta@ncnp.go.jp

**Supplementary Tables**

Mean durations of bradycardia and desaturation in the infants depending on body position or/and sleep status are presented from Supplementary Table S1 to S8.

**Supplementary Table S1. Mean durations of bradycardia among the three body positions throughout the 24-hour recordings**

|  | **Supine** | **Lateral** | **Prone** |
| --- | --- | --- | --- |
|  | **(mean ± s.d.)** | **(mean ± s.d.)** | **(mean ± s.d.)** |
| **HR＜100 bpm (sec)** | **17.1 ± 25.7** | **14.5 ± 25.6** | **8.2 ± 17.8** |
| **HR≧100 bpm (sec)** | **27,554.1 ± 14,072.1** | **26,105.2 ± 13,568.8** | **20,195.1 ± 14,600.0** |

**Supplementary Table S2. Mean durations of desaturation among the three body positions throughout the 24-hour recordings**

|  | **Supine** | **Lateral** | **Prone** |
| --- | --- | --- | --- |
|  | **(mean ± s.d.)** | **(mean ± s.d.)** | **(mean ± s.d.)** |
| **SpO2＜90 (sec)** | **185.2 ± 213.6** | **150.0 ± 174.8** | **54.3 ± 68.3** |
| **SpO2≧90 (sec)** | **27,613.8 ± 14,258.2** | **25,970.4 ± 13,525.2** | **20,149.0 ± 14,576.8** |

**Supplementary Table S3. Mean durations of bradycardia between wake and sleep throughout the 24-hour recordings**

|  | **Wake** | **Sleep** |
| --- | --- | --- |
|  | **(mean ± s.d.)** | **(mean ± s.d.)** |
| **HR＜100 bpm (sec)** | **22.2 ± 31.3** | **17.7 ± 36.2** |
| **HR≧100 bpm (sec)** | **29,113.5 ± 10,766.0** | **44,740.9 ± 13,930.4** |

**Supplementary Table S4. Mean duration of desaturation between wake and sleep throughout the 24-hour recordings**

|  | **Wake** | **Sleep** |
| --- | --- | --- |
|  | **(mean ± s.d.)** | **(mean ± s.d.)** |
| **SpO2＜90**% **(sec)** | **283.0 ± 335.4** | **106.6 ± 149.5** |
| **SpO2≧90**% **(sec)** | **29,081.3 ± 10,228.1** | **44,652.0 ± 13,907.6** |

**Supplementary Table S5. Mean durations of bradycardia among the three body positions during wake**

|  | **Supine** | **Lateral** | **Prone** |
| --- | --- | --- | --- |
|  | **(mean ± s.d.)** | **(mean ± s.d.)** | **(mean ± s.d.)** |
| **HR＜100 bpm (sec)** | **10.2 ± 13.7** | **8.8 ± 16.8** | **3.1 ± 12.0** |
| **HR≧100 bpm (sec)** | **13,159.5 ± 7,413.9** | **10,647.0 ± 5,988.3** | **5,307.0 ± 4,460.3** |

**Supplementary Table S6. Mean duration of bradycardia among the three body positions during sleep**

|  | **Supine** | **Lateral** | **Prone** |
| --- | --- | --- | --- |
|  | **(mean ± s.d.)** | **(mean ± s.d.)** | **(mean ± s.d.)** |
| **HR＜100 bpm (sec)** | **6.9 ± 17.5** | **5.7 ± 16.0** | **5.1 ± 12.7** |
| **HR≧100 bpm (sec)** | **14,394.7 ±11,390.3** | **15,458.2 ± 10,281.8** | **14,888.1 ± 12,391.9** |

**Supplementary Table S7. Mean duration of desaturation among the three positions during wake**

|  | **Supine** | **Lateral** | **Prone** |
| --- | --- | --- | --- |
|  | **(mean ± s.d.)** | **(mean ± s.d.)** | **(mean ± s.d.)** |
| **SpO2＜90**% **(sec)** | **138.5 ± 177.2** | **106.8 ± 156.8** | **37.6 ± 63.9** |
| **SpO2≧90**% **(sec)** | **13,259.3 ± 7,097.3** | **10,549.4 ± 5,943.1** | **5,272.6 ± 4,422.9** |
|  |  |  |  |

**Supplementary Table S8. Mean duration of desaturation among the three positions during sleep**

|  | **Supine** | **Lateral** | **Prone** |
| --- | --- | --- | --- |
|  | **(mean ± s.d.)** | **(mean ± s.d.)** | **(mean ± s.d.)** |
| **SpO2＜90**% **(sec)** | **46.6 ± 88.4** | **43.2 ± 80.4** | **16.8 ± 32.1** |
| **SpO2≧90**% **(sec)** | **14,354.5 ± 11,360.4** | **15,421.0 ± 10,247.3** | **14,876.5 ± 12,386.2** |
